# The Role of Dietary Fiber in Rheumatoid Arthritis Patients: A Feasibility Study

**DOI:** 10.3390/nu11102392

**Published:** 2019-10-07

**Authors:** Julian Häger, Holger Bang, Melanie Hagen, Michael Frech, Pascal Träger, Maria V. Sokolova, Ulrike Steffen, Koray Tascilar, Kerstin Sarter, Georg Schett, Jürgen Rech, Mario M. Zaiss

**Affiliations:** 1 Department of Internal Medicine 3 - Rheumatology and Immunology, Friedrich-Alexander University (FAU) Erlangen-Nürnberg and Universitätsklinikum Erlangen, 91054 Erlangen, Germany; julian.haeger@fau.de (J.H.); Melanie.hagen@uk-erlangen.de (M.H.); Michael.frech@uk-erlangen.de (M.F.); Pascal.Traeger@extern.uk-erlangen.de (P.T.); Maria.sokoloca@uk-erlangen.de (M.V.S.); Ulrike.steffen@uk-erlangen.de (U.S.); Koray.tascilar@uk-erlangen.de (K.T.); Kerstin.Sarter-Zaiss@uk-erlangen.de (K.S.); Georg.Schett@uk-erlangen.de (G.S.); Juergen.rech@uk-erlangen.de (J.R.); 2 Melio.Care GmbH, 91080 Marloffstein, Germany; info@melio.care

**Keywords:** high-fiber diet (HFD), short chain fatty acids (SCFA), rheumatoid arthritis (RA), gut–joint axis, microbiota

## Abstract

Short-chain fatty acids are microbial metabolites that have been shown to be key regulators of the gut–joint axis in animal models. In humans, microbial dysbiosis was observed in rheumatoid arthritis (RA) patients as well as in those at-risk to develop RA, and is thought to be an environmental trigger for the development of clinical disease. At the same time, diet has a proven impact on maintaining intestinal microbial homeostasis. Given this association, we performed a feasibility study in RA patients using high-fiber dietary supplementation with the objective to restore microbial homeostasis and promote the secretion of beneficial immunomodulatory microbial metabolites. RA patients (*n* = 36) under routine care received daily high-fiber bars or cereals for 28 days. Clinical assessments and laboratory analysis of immune parameters in blood and stool samples from RA patients were done before and after the high-fiber dietary supplementation. We observed an increase in circulating regulatory T cell numbers, favorable Th1/Th17 ratios, as well as decreased markers of bone erosion in RA patients after 28 days of dietary intervention. Furthermore, patient-related outcomes of RA improved. Based on these results, we conclude that controlled clinical studies of high-fiber dietary interventions could be a viable approach to supplement or complement current pharmacological treatment strategies.

## 1. Introduction

Rheumatic disorders are among the most prevalent chronic diseases of the musculoskeletal system and connective tissue [1]. The most common inflammatory rheumatic disease, rheumatoid arthritis (RA), is a debilitating, chronic autoimmune disorder affecting approximately 1% of the world population [2,3]. RA is considered a heterogeneous and complex disease where both genetic and environmental risk factors contribute to disease [4]. While several genetic factors have been associated with the risk of RA, among them *major histocompatibility complex* (MHC) class I alleles, in particular DR4 [5] that recognizes the DRB1*04 gene products, the genetic associations alone are insufficient for explaining disease onset, and additional environmental triggers have been proposed.

One important environmental trigger appears to be the diet. Notably, in the last decades, an increase in the incidence of autoimmune and allergic diseases has been documented in developed countries worldwide [6], which coincides with dramatic changes in diet, including reduced fiber intake [7]. Dietary fibers are complex carbohydrates consisting of both soluble and insoluble components. The insoluble fibers have important bulking properties, whereas the soluble forms are fermented by certain species of the gut microbiota, leading to physiologically active metabolites. Short-chain fatty acids (SCFA) are among the most abundant of these active metabolites representing a fuel source for intestinal epithelial cells [8], shaping the gut morphology and function [9], and serving as an energy source for certain bacterial species [10].

Diet affects the diversity of the gut microbiota and thus its secreted metabolites [11,12,13,14]. These findings combined with recent observations in animal models and humans further highlight the importance of the gut–joint axis and make dietary interventional studies—already conducted in the last century—again up to date. Various dietary intervention studies in RA patients have been published in the last century [15,16,17,18,19], including double-blinded, placebo-controlled clinical trials using n-3 fatty acid supplementation, short-term fasting [20,21], vegan, vegetarian, or Mediterranean diets [22,23]. Among those, vegetarian [20] and Mediterranean [23]-based dietary interventions showed an attenuation of disease activity in RA patients. Following a one-year vegetarian diet, Kjeldsen-Kragh et al. report on statistically significant improvements in the number of tender joints, number of swollen joints, pain, and duration of morning stiffness [20]. Also, a strict three-month Mediterranean-based diet in RA patients ensued clinical benefits, which were shown by a significant reduction in DAS28 scores and improved life quality assessed by Health Assessment Questionnaire (HAQ) and SF-36 health surveys compared to RA control patients on standard diets [23]. That is of particular interest, as both the vegetarian and the Mediterranean diet are especially high in fiber, and were shown to significantly increase SCFA levels [13]. Moreover, daily probiotic supplementation of RA patients using capsules filled with 108 colony-forming units of live gut bacteria species of the genus *Lactobacillus casei* for two months exert beneficial effects on arthritis by attenuating DAS28 scores and reducing pro-inflammatory cytokine levels [24]. Once more, the inoculation of probiotic Lactobacillus strains was shown to modulate the gut microbiota and specifically increase SCFA concentrations [25]. Taken together, one might suggest that primarily, those dietary interventions or probiotic supplementation studies that also increase SCFA levels are most effective in attenuating clinical RA symptoms.

Encouraged on recent findings about the immunomodulatory impact of SCFA [26,27,28,29,30,31] and reports specifically highlighting their benefits in inflammatory arthritis and bone homeostasis [32,33,34,35] in mice, we have conducted a short-term feasibility study using high-fiber bar or cereal dietary supplementation in RA patients. Tregs act as immune regulatory cells [36] and beneficially affect bone homeostasis [34,37,38,39], and are considered to malfunction in RA [40]. Therefore, Tregs were used as the primary end-point of our here presented feasibility study in RA patients. We hypothesized, based on own previous work [35,37,38,39] and the Treg-inducing effects of SCFA [26,29], that within our dietary high-fiber supplementation study, increased Treg numbers would most likely beneficially affect arthritis and related bone loss. In order to avoid changing the participating patients’ lifestyle or daily eating behaviors, our study intervention was restricted to basically eating one single high-fiber bar (or equal cereal portion) daily over a 28-day study period. First, this approach was shaped based on the widely articulated desire of RA patients for add-on nutritional treatments to support ongoing therapies disease modifying anti-rheumatic drugs. Second, this approach was based on the ambivalent attitude of patients toward the ingestion of pharmaceutical-made nutritional supplementation. Moreover, a nutritional approach would theoretically allow us to equally and optimally fuel all known SCFA-producing pathways [16] contrasting pharmaceutical forms of direct SCFA supplementation, and enables personalized adaptations with further dietary supplements that may be needed.

## 2. Materials and Methods

### 2.1. Study Subjects

**Figure 1:**
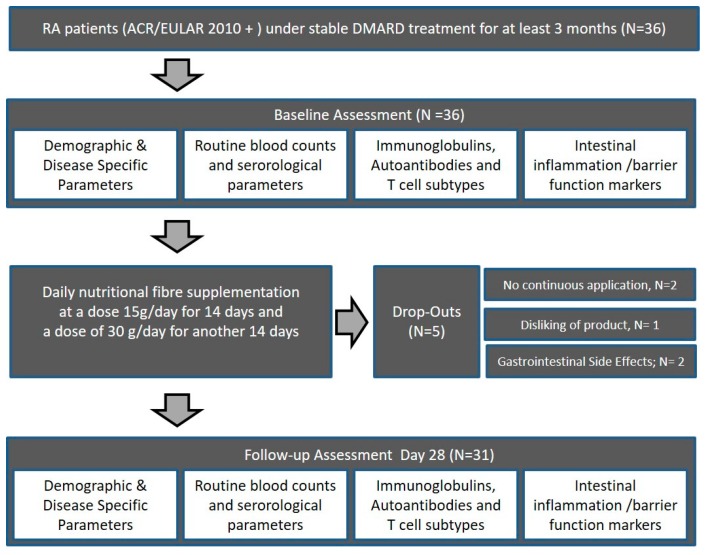
Subject flow chart of the high-fiber dietary supplementation feasibility study. RA, rheumatoid arthritis; DMARDs, disease-modifying anti-rheumatic drugs. ACR/EULAR, American College of Rheumatology/European League Against Rheumatism.

From November 2018 to January 2019, a total of 36 subjects fulfilling the American College of Rheumatology/European League Against Rheumatism (ACR/EULAR) classification criteria for RA, being in clinical remission (Disease Activity Score ≤2.6) were included in this prospective study [41]. After given informed consent, all eligible patients were assessed as consecutive cases. Participants were recruited at the outpatient clinic of the Medical Department 3 of the University Clinic Erlangen, Germany, and gave written informed consent. Patients with RA according to the ACR/EULAR 2010 criteria receiving stable treatment with disease-modifying anti-rheumatic drugs (DMARDs) (conventional synthetic, targeted synthetic, or biological DMARDs) were instructed in detail on the use of the dietary supplement in addition to their regular treatment (Figure 1). All patients stayed on their initial standard medication throughout the feasibility study, and compliance was confirmed weekly based on new patient orders of further high-fiber portions. RA patients’ habitual intake of fiber was assessed using a modified version of the food frequency questionnaire (FFQ) developed for use in the German Health Examination (DEGS) Survey for Adults 2008–2011 [42], but did not change significantly (*p* = 0.5416) within the 28-day study period. All patients received clinical examination at baseline and 28 days, including measurement of parameters of disease activity (DAS28), physical function (HAQ-DI), and quality of life (SF-36). At baseline and 28 days, routine laboratory as well as immunologic parameters were measured as well. In addition, markers of intestinal inflammation, intestinal permeability, and adaptive T cell-related immunity were assessed at baseline and follow-up (summarized in Section 2.5). The study was approved by the Ethics Committee of Medical University Erlangen-Nürnberg (approval number 357_17B).

### 2.2. Intervention

Bakery products account for around 70% of all consumed cereal products; therefore, in an effort to include SFCA in commonly consumed foods, we have produced bars and cereals that contain 50% of dietary fibers and a wide range of prebiotic ingredients to support the production of SCFA as much as possible. According to the FDA rules ((21 CFR 101.9(c)(6)(i)) for “dietary fiber”, we have added non-digestible soluble and insoluble carbohydrates (with three or more monomeric units) to have physiological effects that are beneficial to human health. In a first in-men study with healthy volunteers, we had selected the following dietary fiber mixture as very effective in improving SCFA production in humans: ground flaxseed, oat flakes, psyllium husk, inulin, arrowroot flour, guar gum, coconut, and hemp flour. Additionally, 0.5% cinnamon was added as a non-synthetic chemical additive for food preservation, achieving a generally accepted taste. The ingredients were mixed according to the standards process of a certified bakery. After baking the bars and cereal, materials were divided into 30-g portions and stored in vacuum packaging to ensure a longer shelf life of the baked product.

### 2.3. Assessment of Safety (Adverse Events, Concomitant Medication, and Tolerability)

All adverse events and concomitant medication were documented during the study period. Tolerability was assessed at the end of the study. Subjects rated the overall tolerability in three categories as “well tolerated”, “slightly unpleasant”, or “very unpleasant”.

### 2.4. Data analysis and Statistics

Clinical and demographic characteristics of patients were summarized and tabulated using appropriate descriptive statistics. The aim and primary end-point of our here presented feasibility study in RA patients was to examine the effect of short-term high-fiber dietary supplementation on Treg numbers. Based on previous data [33] and published data [29] a conservative assumption with an 8% increase in Tregs was applied for the prior sample size calculation, resulting in an effect size of d = 0.828. Based on the following input details—alpha error problem of = 0.05, actual power of 80%—a sample size of *n* = 26 subjects was estimated. We used the Wilcoxon signed-rank test to compare changes in T-cell subpopulations and clinical outcomes between baseline and after the dietary intervention. We did not make adjustments for multiple testing in this exploratory feasibility study. Two-sided *p* values of <0.05 were considered significant, and were shown as *p* * = *p* < 0.5; *p* ** = *p* < 0.01; and *p* *** = *p* < 0.001.

### 2.5. Sample Collection and Processing

#### 2.5.1. Cell Handling and Cryoconservation

Mononuclear cells (MNC) from whole blood samples were isolated by density gradient centrifugation (Pancoll, Pan Biotech) according to the manufacturer’s instructions. MNC were washed in phosphate-buffered saline (PBS) and resuspended in cryoconservation medium (10% dimethyl sulfoxide (DMSO), 90% fetal calf serum (FCS; c.c.pro)) and cryoconserved at 200 × 10^6^/mL over liquid nitrogen for more than 48 h. MNC numbers were determined by a Neubauer cell-counting chamber.

#### 2.5.2. Flow Cytometry

Cells were recovered from cryoconservation in corresponding pairs and washed in cell culture medium (RPMI 1640, Pan Biotec) supplemented with 10% FCS. Prior to staining with antibodies, a LIVE/DEAD Fixable Violet Dead Cell Stain Kit (Life Technologies) was used to exclude dead cells. For intracellular staining, total lymphocytes were incubated in vitro overnight with T-Bet and RORγt antibodies. Antibodies directly coupled with fluochromes were used. Tregs were gated on CD4^+^CD25^+^FoxP3^+^ cells, Th1 cells as CD4^+^T-bet^+^, and Th17 cells as CD4^+^RORγt^+^. All flow cytometry analyses were performed using the CytoFLEX Platform (Beckman Coulter) and analyzed using Kaluza analysis software (Beckman Coulter).

#### 2.5.3. Enzyme-linked Immunosorbent Assay (ELISA)

Cross-Laps CTX-I (IDS, Immunodiagnostic Systems, UK), osteocalcin (OCN) (Invitrogen, Germany), and Zonulin (Cusabio Technology, US) serum ELISA were performed according to the manufacturer’s instructions. Concentrations of total serum immunoglobulin A (IgA), IgA1, and IgA2 were determined by Elisa using goat F(ab)2 anti-human IgA (#2052-01) as capture and HRP-coupled goat anti-IgA (#2050-05), mouse anti-human IgA1 (#9130-05), or mouse anti-human IgA2 (#9140-05) as detection antibodies (all Southern Biotech). Anti citrullinated protein antibodies (ACPA) levels were determined using cyclic-citrullinated peptide (CCP) coated plates (Orgentec Diagnostika). For sera calprotectin ELISA, a sandwich-type method was used based on a coating of 0.5 µg/mL monoclonal antibodies against human calprotectin (Medix Biochimica, Finnland; Orgentec, Mainz, Germany, for research-only kit,), on a standard microtiterplate (Thermo Scientific, Denmark) at 4 °C for 8 h at least, and unspecific binding was blocked with PBS supplemented with 1% of bovine serum albumin. Patient samples were diluted 1:100 in PBS/0.05% and incubated for 30 min. In the presence of calprotectin, a sandwich complex form is made up of immobilized antibodies, calprotectin, and peroxidase-conjugated antibodies. The washing procedure and substrate reaction were performed as described above for the modified vimentin peptides ELISAs.

#### 2.5.4. Stool Sample Processing

Stool samples had been frozen for a maximum of 24 weeks and were thawed immediately before use. Stool Extraction Tubes (ORGENTEC Diagnostika) provided with a universal extraction medium were used according to the manufacturer’s instructions. The tube was filled with 750 μL of extraction medium. The integrated spatula in the closure of the tube was used to pick exactly 15 mg of stool, resulting in a sample dilution of 1:50. The tube was vortexed to dissolve the stool completely in the extraction medium, and the sample was further homogenized for 15 min on a rocking shaker. Then, the homogenate was transferred to a fresh microtube and centrifuged at 3000× *g* for two minutes. The clear supernatant was transferred to a fresh tube, and aliquots of 10 µL were tested immediately for modified vimentin assays.

#### 2.5.5. Modified Vimentin 58-GRVYATRSSAVR-69 (p18)

Patient-processed stool samples were diluted 1:100 in PBS/0.05% Tween 20/1% albumin and incubated for 30 min. Unbound antibodies were washed out with twice with 200 µL of PBS/0.05% Tween. Bound autoantibodies were detected with horseradish peroxidase conjugated anti-human IgG (Dianova, Hamburg, Germany) and visualized with 3,3’, 5,5”-tetramethylbenzidine (TMB) as substrate for the peroxidase. Optical density was measured with standard micro-titer plate reader (Tecan, Germany). Standard curves were established by using a patient serum from the Outpatients Department of Rheumatology, Dresden. The reference range was defined by healthy volunteers (*n* = 300) as mean antibody reactivity plus three standard deviations (SD). The detection of rheumatoid factor IgM, and anti-CCP IgG antibodies were measured using commercially available ELISA kits (Orgentec Diagnostika, Germany), and a cut-off of 20 IU was used to define antibody positivity. In the citrullinated, acetylated, and carbamylated vimentin peptide assays, a cut-off of 20 IU was used to define antibody positivity. Peptides were manufactured and used in accordance with the general protocol for the Orgentec immunometric enzyme immunoassay [43,44]. Briefly, 0.5 µg/mL of biotinylated vimentin peptide either citrullinated (GRVYAT-Cit-SSAVR, carbamylated (GRVYAT-HCit-SSAVR), or acetylated (GRVYAT-Lys(ac)-SSAVR were loaded on streptavidin precoated cavities of a standard micro-titerplate (Thermo Scientific, Denmark), and unspecific binding was blocked with PBS supplemented with 1% bovine serum albumin.

## 3. Results

### 3.1. Patient Charactristics

Demographic data and the current medication of participating patients are summarized in Table 1.

### 3.2. Effects on T cell Homeostasis

**Figure 2:**
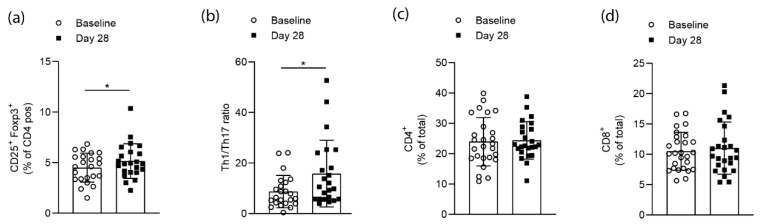
Short-term high-fiber supplementation effects on T-cell populations of RA patients. (**a**) Flow cytometry analysis of CD4^+^CD25^+^FoxP3^+^ cells from whole blood samples of RA patients before and after high-fiber supplementation (*p* = 0.0138); (**b**) Th1/Th17 ratios for each participating RA patient calculated based on flow cytometry analysis for CD4^+^T-bet^+^ (Th1) and CD4^+^RORγt^+^ (Th17) cells (*p* = 0.0135); (**c**) CD4^+^ and (**d**) CD8^+^ T cells from whole blood samples in RA patients. Data is expressed as mean ± SD. Statistical difference was determined by Wilcoxon matched-pairs signed-rank test.

Flow cytometry analysis of CD4^+^CD25^+^FoxP3^+^ cells from whole blood samples of RA patients at baseline and after the 28 days of high-fiber supplementation revealed a significant (*p* = 0.0138) increase in the numbers of Tregs at day 28 (Figure 2a). Flow cytometry analysis for CD4^+^T-bet^+^ (Th1) and CD4^+^RORγt^+^ (Th17) cells following high-fiber supplementation revealed a significant (*p* = 0.0135) increase of the Th1/Th17 ratio on day 28 (Figure 2b). At the same time, neither total CD4^+^ or CD8^+^ T-cell numbers were changed (Figure 2c,d). Hence, high-fiber supplementation appears to affect T-cell polarization rather than globally affecting T-cell numbers.

### 3.3. Effects on Signs and Symptoms of Arthritis

As RA patients do not have substantial inflammatory activity at baseline due to stable effective DMARD therapy, disease activity measures such as DAS 28 were low already at baseline, and did not significantly change during the study period (Table 2). However, the Short Form (SF) 36 Health Survey, which is a 36-item instrument for measuring patients’ quality of life, significantly improved after the 28-day period of nutritional intervention affecting both the physical (physical functioning, *p* = 0.0007) and the mental components of life quality (general health, *p* = 0.0137) (Table 2). The Health Assessment Questionnaire Disability Index (HAQ-DI) is currently the most widely used measure of physical functioning and disability across rheumatic diseases, particularly in RA [45]. Here, within this short period of dietary high-fiber supplementation, HAQ values did also show significant (*p* = 0.0036) improvements (Table 2).

### 3.4. Effects on Routine Laboratory Parameters and Markers of Bone Resorption

Routine laboratory assessments did not show a significant change during the short study period (Table 3). There was a slight increase in blood eosinophil counts at day 28 (*p* = 0.0558) (Table 3). C-reactive protein levels were low at baseline, and slightly but not significantly (*p* = 0.2246) decreased after 28 days. Chronic inflammation in RA also leads to bone loss. Bone destruction is caused by an elevated resorption activity of osteoclasts, which can be measured by increased levels of collagen fragments (CTX-I) [46]. Here, we show that serum CTX-I (Crosslaps) concentrations were significantly reduced (*p* = 0.0322) in RA patients treated with high-fiber supplementation (Table 4).

### 3.5. Effects on Immunological Parameters

We observed significant changes in the levels of mucosal immunity-associated IgA subclasses after nutritional intervention (Table 4). Hence, the total IgA (*p* = 0.034) and IgA1 (*p* = 0.0442) were significantly reduced at 28 days following high-fiber supplementation. The effects of IgA were more global rather than specific for IgA antibodes against citrullinated proteins. The IgM rheumatoid factor and IgG anti-citrullinated protein antibodies, which are rather specific for RA-related autoimmunity and associated with more severe disease [41,47] slightly decreased, but overall were not significantly changed during the 28-day treatment. Of note, anti-citrullinated vimentin p18 peptide antibody levels were also significantly (*p* = 0.0063) reduced 28 days after high-fiber supplementation (Table 4).

### 3.6. Effects on Intestinal Markers of Inflammation and Barrier Function

Moreover, we observed significantly reduced serum calprotectin (*p* = 0.0039) and zonulin (*p* = 0.0407) levels after high-fiber dietary intervention (Table 4).

## 4. Discussion

Most dietary fibers are fermented by gut bacteria, and thus give rise to the microbial metabolites SCFA. The three main SCFA detected in our bodies are acetate, propionate, and butyrate. A decreased intake of dietary fibers and increased intake of fat and sugar in our food, which is typical for a Western lifestyle, were shown to contribute to intestinal microbial dysbiosis by the depletion of specific bacterial taxa of the gut microbiota [48]. Microbial dysbiosis in the gut was shown to promote severe immunological dysfunctions [49] that in turn may contribute to the immune dysbalance observed in RA [36]. In this context, it was proposed that inflammatory diseases may, at least in part, be affected by dietary fiber supplementation, arguing for attempts to overcome the ‘‘fiber gap’’ through selective fiber-rich adjustments in our diet [50].

We have previously shown that a high-fiber diet or direct SCFA supplementation can attenuate experimental arthritis and inhibit bone loss [33,35]. At the same time, microbial dysbiosis has been associated with arthritis, pointing toward a gut–joint axis in the onset of arthritis. For example, as early as the 1970s, it was shown that intestinal infections frequently preceded the occurrence of clinical arthritis [51]. Later, Kohashi et al. demonstrated in 1979 that germ-free (GF) rats develop a particularly severe form of RA in an adjuvant-induced arthritis model [52]. Other studies have shown the effects of both Gram-positive or Gram-negative intestinal bacteria on arthritis, further suggesting that the gut microbiota has a significant influence on the development of RA [52,53]. Additional data showed that the intestines of germ-free HLA-B27 rats or B10.BR (h-2 (k)) mice—these animal models are predisposed to develop spontaneous arthritis—following selective repopulation with different bacterial strains were even more prone to develop arthritis [54,55]. These data were recently complemented by showing that the onset and severity of clinical arthritic symptoms in genetically susceptible mice depends on the gut microbiota composition [56,57,58].

In an effort to translate these observations obtained in animal models to humans, 16s rRNA sequencing methods were used in order to correlate individual bacterial strains within the gut microbiota and the onset of clinical signs in human RA patients [59,60]. These data showed that commensal gut bacteria belonging to the Prevotellaceae family are enriched in RA patients, especially in pre-clinical stages of RA [61,62,63,64]. However, discrepancies in the pro-arthritogenic and anti-arthritogenic effects of specific gut microbes used in animal models, together with the so-far indistinct results of human 16s rRNA microbiota sequencing, suggest that there may be no single gut bacterial strain that is promoting or inhibiting arthritis on its own. In contrast, distinct bacterial communities or their secreted bacterial metabolites may function as environmental triggers responsible for the onset or prevention of arthritis. Taken together, existing human studies strengthen the gut–joint axis by identifying a microbial dysbiosis early in human arthritis that is partially normalized during the treatment of arthritis [65].

Although attenuating effects on RA pathology were observed in dietary intervention studies, none of them has been successfully implemented in the clinics so far. The reasons for that might be: (i) a scarcity of acceptance and persuasion of rheumatologists on this topic, (ii) unidentified underlying immunological mechanisms explaining the improvement in RA patients, or (iii) the existence of therapeutically very effective DMARDS that make dietary alternatives less prominent. However, all dietary intervention therapies require changes to the patients’ daily lifestyle and eating behaviors, and that requires commitment, endurance, persuasion, and time. Therefore, in the current study, we considered a simple dietary supplementation using high-fiber bars or cereals to close the “fiber gap”, while leaving the standard nutrition of each individual untouched.

The aim and primary end-point of our here presented feasibility study in RA patients was to examine the effect of short-term high-fiber dietary supplementation on Treg numbers. We hypothesized that dietary supplementation with fiber can strengthen regulatory immune elements, thereby helping to control and even suppress disease progression in RA, and in consequence inhibit bone loss and the impairment of function. Tregs, which act as immune regulatory cells [36] and beneficially affect bone homeostasis [34,37,38,39] are considered to malfunction in RA [40]. Therefore, Treg-supporting effects, as observed in our dietary high-fiber supplementation study, are the most likely to beneficially affect arthritis and related bone loss. Alongside the increased Treg numbers observed in our feasibility study, RA patients also showed beneficial Th1/Th17 cell ratios, lowered citrullinated vimentin peptide 18 and serum IgA concentrations, and improved parameters for quality of life, as shown by the HAQ and SF36 questionnaire outcomes. It was shown previously that RA patients exhibit a decreased Th1/Th17 ratio compared to healthy individuals [66,67]. Interestingly, dietary high-fiber supplementation, as shown here in our feasibility study, skewed the ratio in the opposite direction, initiating anti-arthritic processes. While the impact of IgG autoantibodies on the development of RA has been intensively studied, the role of IgA autoantibodies is still less well defined, but potentially more relevant for understanding intestinal function [68]. Synovial biopsies of RA patients revealed that up to 38% of plasma cells produced IgA [69], and subjects at risk for developing RA showed an increased percentage of IgA-producing peripheral blood plasmablasts [70]. These data suggest that reducing serum IgA levels in RA patients may represent another health-promoting effect observed in our feasibility study. In addition, citrullinated vimentin was shown to be highly abundant in RA synovial tissues [71], and among the possible 43 putative citrullinated arginine residues, the citrullinated vimentin 58-GRVYATRSSAVR-69 (p18) peptide had one of the highest reactivities with RA sera [72]. An epitope spreading of RA-related immunity against citrullinated peptides has been observed in the evolution of RA [73,74], while data from our here presented study suggest that some of these changes may indeed be at least partially reversible, as shown for reduced p18 concentrations, and that dietary fiber supplementation is a key driver of this reversal.

Calprotectin is a 24-kDa dimer of calcium-binding proteins S100A8 and S100A9. The measurement of fecal calprotectin has emerged as one of the most useful tools for quantifying intestinal inflammation [75], and zonulin controls intestinal permeability and disintegrates intestinal tight junctions [76,77]. Short-term dietary high-fiber supplementation clearly supported gut homeostasis by reducing the intestinal inflammation marker, calprotectin [72] and the intestinal barrier marker zonulin [69] in our feasibility study in RA patients. Zonulin expression has been recently shown to be augmented in autoimmune conditions associated with tight junction dysfunction, including celiac disease (CD) and Type 1 diabetes (T1D) [78,79]. Interestingly, both animal studies [80] and human trials [81] using the zonulin synthetic peptide inhibitor AT1001 (Larazotide acetate) established that zonulin is integrally involved in the pathogenesis of autoimmune diseases. Therefore, decreased zonulin levels by dietary high-fiber supplementation might be a valuable additional beneficial therapeutic effect for RA patients. Following our high-fiber supplementation period, we also observed a tendency (*p* = 0.0558) toward higher eosinophil numbers. This is of potential further interest, especially with respect to a recent study highlighting a beneficial effect of eosinophils in animal models of arthritis [82], whereas the translational proof in RA patients is still missing to date.

As a limitation of this first short-term feasibility study, RA patients were recruited independently of their current treatment, not showing substantial inflammatory activity at baseline due to stable effective therapies. In addition, no control group was included. Hence, future follow-up studies should include new-onset RA and active RA patients, taking into account different DMARD therapies as well as longer, up to three-month dietary supplementation periods using a randomized cross-over design. A further limitation is that easy-to-use high-fiber supplementation bars or cereals yet need to be improved for stability and optimal daily dosage. On the other hand, the here presented data from our first high-fiber dietary supplementation feasibility study in RA patients clearly showed the potential of nutritional medicine in supporting ongoing standard therapies.

In summary, in this short-term high-fiber diet-based dietary supplementation feasibility study, the physical functioning and quality of life in RA patients was significantly improved, correlating with improved markers of intestinal homeostasis, thereby establishing a stage of immune tolerance by increased Treg numbers in patients with RA. However, these findings should be regarded as preliminary and interpreted with caution, since baseline versus follow-up comparisons in a single-arm study do not necessarily prove a causal treatment effect.

## 5. Conclusions

Our results lay the foundation for further controlled studies on high-fiber supplementation in RA patients in the future.

## Figures and Tables

**Table 1:** Demographic data and current treatment of participating patients.

**Demographic characteristics (N = 36)**	
Age, mean ± SD, years	56.19 ± 7.7
Females, N (%)	20 (64.51%)
BMI, mean ± SD, units	26.63 ± 6.4
Ever smoker, N (%)	6 (19.35%)
**Disease-specific characteristics (N = 36)**	
Disease duration, mean ± SD, years	11.64 ± 9.39
Disease activity score (DAS) 28, mean ± SD, units	2.54 ± 0.28
Anti-CCP-IgG antibody positive, N (%)	15 (48.38%)
Rheumatoid Factor IgM positive, N (%)	12 (38.7%)
**Concomitant anti-rheumatic treatment (N = 36)**	
Methotrexate, N (%)	16 (51.61%)
Glucocorticoids, N (%)	5 (16.12%)
Other csDMARDS, N (%)	1 (3.22%)
**Biological DMARDs**, N (%)	23 (74.10%)
Tumor Necrosis Factor Inhibitors, N (%)	5 (16.12%)
Tocilizumab, N (%)	8 (25.8%)
Abatacept, N (%)	3 (9.67%)
Rituximab, N (%)	7 (22.58%)
JAK-inhibitors, N (%)	4 (12.9%)

BMI: body-mass index, CCP: cyclic-citrullinated peptide, DMARD: disease-modifying anti-rheumatic drug, csDMARD: conventional synthetic DMARD, JAK: Janus kinase, SD: standard deviation.

**Table 2:** Clinical assessment of participating patients.

	Baseline	Day 28
**Disease Activity**		
Disease activity score (DAS) 28, mean ± SD, units	2.56 ± 0.28	2.53 ± 0.28
Swollen joint count, mean ± SD, N	0.61 ± 0.23	0.50 ± 0.22
Tender joints, mean ± SD, N	2.29 ± 0.60	2.25 ± 0.62
Visual analogue scale (VAS) for pain, mean ± SD, cm	25.54 ± 4.78	26.96 ± 4.74
VAS for patients’ global disease activity, mean ± SD, cm	24.54 ± 4.22	26.18 ± 4.25
VAS for physicians´global disease activity, mean ± SD, cm	20.31 ± 4.26	17.85 ± 4.08
**Physical Function**		
Health Assessment Questionaire (HAQ), mean ± SD, units	0.54 ± 0.08	0.43 ± 0.09 **
**Quality of Life**		
SF 36- physical functioning	66.37 ± 5.30	73.74 ± 4.79 ***
SF 36- role limitation/physical, mean ± SD, units	57.69 ± 8.52	58.65 ± 8.54
SF 36- role limitation/emotional, mean ± SD, units	64.04 ± 7.84	60.23 ± 8.68
SF 36- energy/fatigue, mean ± SD, units	55.77 ± 4.60	58.27 ± 4.33
SF 36- emotional wellbeing, mean ± SD, units	70.96 ± 3.96	71.20 ± 3.94
SF 36- social functioning, mean ± SD, units	76.10 ± 4.38	75.16 ± 4.08
SF 36- pain, mean ± SD, units	63.48 ± 3.69	61.87 ± 3.91
SF 36- general health, mean ± SD, units	40.37 ± 3.58	50.53 ± 4.47 *
SF 36- health change, mean ± SD, units	61.00 ± 4.58	55.00 ± 4.08

SD: standard deviation, SF 36: short form 36. *p* * = *p* < 0.5; *p* ** = *p* < 0.01; and *p* *** = *p* < 0.001.

**Table 3:** Blood values of participating patients.

Laboratory Tests	Baseline	Day 28
C-reactive protein, mean ± SD, mg/L	6.74 ± 0.61	6.01 ± 0.36
Erythrocytes, mean ± SD, N × 10^6^/µL	4.60 ± 0.07	4.60 ± 0.07
Hematocrit, mean ± SD, %	41.25 ± 0.52	40.96 ± 0.49
Leukocytes, mean ± SD, N × 10^3^/µL	6.41 ± 0.46	6.50 ± 0.50
Thrombocytes, mean ± SD, N × 10^3^/µL	250.2 ± 10.65	262.2 ± 11.83
Neutrophils, mean ± SD, N × 10^3^/µL	4.07 ± 0.43	3.83 ± 0.36
Monocytes, mean ± SD, N × 10^3^/µL	0.54 ± 0.03	0.52 ± 0.08
Lymphocytes, mean ± SD, N × 10^3^/µL	1.54 ± 0.10	1.63 ± 0.11
Eosinophils, mean ± SD, N × 10^3^/µL	0.13 ± 0.01	0.16 ± 0.02
Basophils, mean ± SD, N × 10^3^/µL	0.04 ± 0.01	0.04 ± 0.01
Creatinin, mean ± SD, mg/dL	0.76 ± 0.03	0.75 ± 0.03
Uric acid, mean ± SD, mg/dL	5.28 ± 0.22	5.19 ± 0.25
Sodium, mean ± SD, mmol/ L	139.8 ± 0.28	140.0 ± 0.33
Potassium, mean ± SD, mmol/L	4.41 ± 0.08	4.42 ± 0.07
LDH, mean ± SD, U/L	274.3 ± 12.10	264.2 ± 8.81
Creatine kinase, mean ± SD, U/L	130.2 ± 16.28	158.3 ± 29.53
ALT, mean ± SD, U/L	26.42 ± 2.94	29.04 ± 2.77
Alkaline phosphatase, mean ± SD, U/L	78.39 ± 3.96	81.52 ± 4.69
LDL- cholesterol, mean ± SD, mg/dL	160.3 ± 7.17	153.5 ± 7.53
HDL- Cholesterol, mean ± SD, mg/dL	70.31 ± 2.39	68.94 ± 3.48
Triglycerides, mean ± SD, mg/dL	105.9 ± 10.51	94.38 ± 8.86
Hba1c, mean ± SD, %	5.50 ± 0.11	5.51 ± 0.11

SD: standard deviation, LDH: lactate dehydrogenase; LDL: low density lipoprotein, HDL: high density lipoprotein, Hba1c: glycated hemoglobin 1c.

**Table 4:** Serum parameters of participating patients.

Immunological Parameters	Baseline	Day 28
RF- IgM, mean ± SD, Units	211.9 ± 98.26	185.7 ± 79.35
RF IgA, mean ± SD, Units	142.4 ± 62.80	127.9 ± 50.85
ACPA-IgG (CCP2 test), mean ± SD, Units	231.2 ± 76.94	170.7 ± 53.21
IgA1, mean ± SD, OD	3911 ± 537.5	3599 ± 515.1 *
IgA2, mean ± SD, OD	215.2 ± 32.88	211.9 ± 39.06
Total IgA, mean ± SD, OD	4206 ± 558.9	3873 ± 538.7 *
ACPA (CCP2 test) IgA1, mean ± SD, µg/mL	3.12 ± 0.36	2.83 ± 0.35
ACPA (CCP2 test) IgA2, mean ± SD, µg/mL	0.68 ± 0.10	0.64 ± 0.10
Total ACPA IgA, mean ± SD, µg/mL	4.08 ± 0.31	3.78 ± 0.37
Anti-citrullinated VIM p18, mean ± SD, OD	654.5 ± 116.4	460.3 ± 89.25 **
Anti- Acetylated ornithine VIM p18, mean ± SD, OD	311.0 ± 100.7	303.7 ± 107.3
Carbamylated vimentin VIM p18, mean ± SD, OD	284.6 ± 126.4	229.3 ± 97.45
Acetylated lysine VIM p18, mean ± SD, OD	299.0 ± 141.8	276.4 ± 132.3
Calprotectin, mean ± SD, ng/mL	6.06 ± 0.71	4.52 ± 0.31 **
Zonulin, mean ± SD, ng/mL	4.01 ± 0.51	2.91 ± 0.32 *
Crosslaps, mean ± SD, ng/mL	0.42 ± 0.05	0.36 ± 0.05 *
Osteocalcin, mean ± SD, ng/mL	16.31 ± 1.16	16.73 ± 1.40

RF: rheuma factor, SD: standard deviation, OD: optical density, Ig: immunoglobulin, ACPA: anti citrullinated protein antibodies. *p* * = *p* < 0.5; *p* ** = *p* < 0.01.
